# Melatonin Protects against Lung Fibrosis by Regulating the Hippo/YAP Pathway

**DOI:** 10.3390/ijms19041118

**Published:** 2018-04-09

**Authors:** Xiaoguang Zhao, Jian Sun, Wei Su, Huitong Shan, Bowen Zhang, Yining Wang, Azaliia Shabanova, Hongli Shan, Haihai Liang

**Affiliations:** 1 Department of Pharmacology (State-Province Key Laboratories of Biomedicine-Pharmaceutics of China, Key Laboratory of Cardiovascular Research, Ministry of Education), College of Pharmacy, Harbin Medical University, Harbin 150081, China; zhaoxiaoguang@hrbmu.edu.cn (X.Z.); sunjian@hrbmu.edu.cn (J.S.); suwei@hrbmu.edu.cn (W.S.); shanhuitong@hrbmu.edu.cn (H.S.); zhangbowen@hrbmu.edu.cn (B.Z.); wangyining@hrbmu.edu.cn (Y.W.); azaliias@yahoo.com (A.S.); shanhongli@ems.hrbmu.edu.cn (H.S.); 2 Northern Translational Medicine Research and Cooperation Center, Heilongjiang Academy of Medical Sciences, Harbin Medical University, Harbin 150081, China; 3 Department of Outpatient and Emergency Pediatric, Bashkir State Medical University, Ground Floor, Teatralnaya Street, 2a, 450000 Ufa, Russia

**Keywords:** idiopathic pulmonary fibrosis, melatonin, YAP1

## Abstract

Idiopathic pulmonary fibrosis (IPF) is a progressive, fibrotic interstitial pneumonia with high mortality. Melatonin, a hormone predominantly secreted by the pineal gland, has been reported to participate in the process of IPF. However, the mechanisms underlying the effect of melatonin in pulmonary fibrosis have not been elucidated to date. This study was designed to evaluate the anti-fibrotic role of melatonin in pulmonary fibrosis and to elucidate the potential mechanisms. We observed that melatonin markedly attenuated bleomycin (BLM)-induced experimental lung fibrosis in mice and inhibited TGF-β1-induced fibrogenesis in lung fibroblasts. Additionally, we determined that luzindole, a melatonin receptor inhibitor, reduced the anti-fibrotic effect of melatonin. Further studies showed that melatonin alleviated the translocation of YAP1 from cytoplasm to nucleus, a key downstream effector of the Hippo pathway, in vivo and in vitro by interacting with its receptor. Taken together, our results suggest that melatonin prevents lung fibrosis by inhibiting YAP1 and indicate that melatonin replacement could be a novel strategy for the treatment of lung fibrosis.

## 1. Introduction

Idiopathic pulmonary fibrosis displays a condition in which the natural lung biological makeup is replaced by progress of lung structure aberrant remodeling, abundant accumulation of extracellular matrix and dramatic changes in the cell phenotype of both fibroblasts and alveolar epithelial cells [1]. IPF, characterized by chronic, devastating and fibrotic interstitial pneumonia, contributes to the derangement of the respiratory function, eventually resulting in death. Therapeutic options are limited by a poor understanding of the underlying pathogenesis, although nintedanib and prifenidone could ameliorate the lung fibrosis to a certain extent [2]. Herein, it is imminent to explore the underlying molecular basis of IPF and to furnish adequate theoretical foundations for the clinical treatment.

Melatonin (*N*-acetyl-5-methoxytryptamine) is a neurohormone primarily secreted from the pineal gland [3,4]. The functional effect of melatonin involves a wide spectrum of pathophysiological processes, including delaying senescence [5], anti-tumorigenesis [6], sleep melioration [7] and immune adjustment [8]. The function of melatonin is mainly mediated by its receptors, MT1 and MT2, classical G protein-coupled receptors [9,10]. Furthermore, growing evidence suggests that melatonin exerts anti-fibrotic effects in the heart, kidney, lung, liver and other organs [4]. During the pathogenesis of fibrosis, melatonin plays a protective role in initial injury to the organ, activation of effector cells (fibroblasts, myofibroblasts, and inflammatory cells), and accumulation of ECM. Martinez et al. [11] demonstrated that melatonin inhibited the leptin-induced augmentation of Collagen1 and up-regulation of fibrotic markers, such as fibronectin, connective tissue growth factor (CTGF), and transforming growth factor β (TGF-β) in cardiac myofibroblasts. Das et al. [12] found that melatonin mitigated hepatic fibrosis in mice by modulating hepatic stellate cell activation. Additionally, melatonin alleviated bleomycin (BLM)-induced pulmonary fibrosis via suppression of lipid peroxidation [13]. Although an increasing number of studies have revealed the significant role of melatonin in the pathophysiological processes of pulmonary fibrosis, an exhaustive examination of the detailed mechanisms has not been presented.

Recent studies have shown that the Hippo signaling pathway plays critical roles in a variety of pivotal pathological processes, including organ growth control, cell proliferation, apoptosis, tissue regeneration and tumor suppression [14,15]. Yes-associated protein (YAP), a key downstream effector of Hippo, has aroused great interest in studies of human diseases. Zhang et al. reported that OroxylinA ameliorated angiogenesis in liver fibrosis by inhibiting Hippo-YAP signaling [16]. However, the effect of the Hippo pathway in IPF is still unclear. Recently, it was reported that GPCR signals participate in regulation of the Hippo signaling pathway [17,18]. As mentioned above, melatonin signal transduction is mainly dependent on the function of MT1 and MT2. Therefore, we hypothesized that MT1 and MT2, which are classical GPCRs, activate the Hippo signal cascade to exert the anti-fibrotic function of melatonin during the process of IPF.

In this study, we found that melatonin attenuated TGF-β1-induced fibrogenesis in lung fibroblasts by activating the Hippo pathway and later promoting the nuclear translocation and increasing the inactivation and degradation of YAP1 in the cytoplasm. Furthermore, we also revealed that melatonin mitigated experimental pulmonary fibrosis in mice treated with BLM. This study elucidated the potential role of melatonin in the development of IPF, which may open therapeutic avenues for drug discovery and treatment of pulmonary fibrosis.

## 2. Results

### 2.1. Protective Role of Melatonin in BLM-Induced Lung Fibrosis in Mice

**Figure 1:**
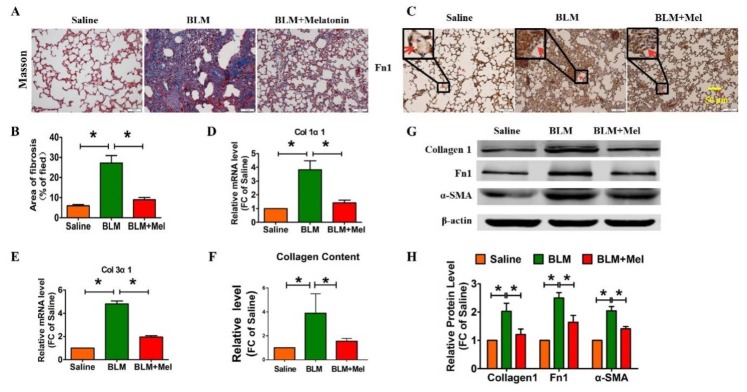
Melatonin alleviates bleomycin-induced pulmonary fibrosis in mice. (**A**) Representative microscopy images of Masson’s staining of lung tissue derived from BLM-treated mice or BLM + Melatonin mice; scale bar, 50 μm; (**B**) Quantification of total interstitial fibrotic area using Image-Pro Plus; (**C**) IHC staining shows the differential expression of fibronectin 1 (Fn1). *n* = 6 mice in each group, red arrow indicated the expression of Fn1. The mRNA expression of Col 1α1 (**D**) and Col 3α1 (**E**) was measured via qRT-PCR. β-Actin mRNA acted as the internal control; (**F**) Collagen content of lung tissues was detected by SircolTM Soluble Collagen Assay; (**G**) Western blot analysis examining the fibrotic-related protein expression in BLM-treated mice with or without melatonin injection; (**H**) The statistical Western blotting data. *n* = 4 mice in each group. Values are the mean ± SEM of five independent experiments. * *p* < 0.05.

To investigate whether melatonin participates in the progression of idiopathic pulmonary fibrosis, we constructed an animal model via intratracheal injection of BLM [19], along with or without intraperitoneal injection of melatonin (5 mg/kg/d). Four weeks later, we evaluated the formation of fibrotic foci and fibrosis-related markers. Masson’s trichrome staining suggested that the fibrosis mouse model was successfully established, and melatonin significantly alleviated the BLM-induced pulmonary fibrosis in mice (Figure 1A,B). In addition, immunohistochemistry assays revealed that melatonin reduced the expression of fibronectin1 (Fn1) in BLM-induced mice (Figure 1C). The mRNA level of collagen 1α1 (Col1α1) and collagen 3α1 (Col3α1) were measured via real-time RT-PCR, and the protein level of fibrotic-related markers were detected with Western blotting. As shown in Figure 1D,F, melatonin significantly attenuated the expression of Col1α1 and Col3α1 at the mRNA level and abolished the increased collagen deposition induced by BLM. Moreover melatonin inhibited and the expression of fibrosis-related genes at the protein level, such as collagen 1, and Fn1 (Figure 1G,H). Hence, these data revealed that melatonin ameliorated pulmonary fibrosis in vivo.

### 2.2. Melatonin Attenuates Pulmonary Fibrosis by Interacting with Its Specific Receptors

**Figure 2:**
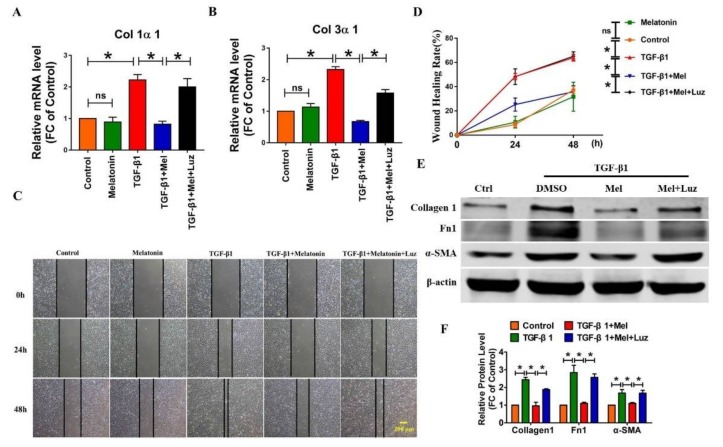
Identification of melatonin receptor as a functional target in melatonin-mediated fibrosis. mRNA expression of Col 1α1 (**A**) and Col 3α1 (**B**) was obtained with qRT-PCR. β-Actin was used as the internal control. (**C**,**D**) Wound healing assay demonstrated that luzindole abolished the TGF-β1-induced cell migration and the Western blot (**E**,**F**) analysis of fibrosis-related proteins illustrated that luzindole blocked the inhibitory effect of melatonin in TGF-β1-induced fibrogenesis. β-Actin served as the loading control. Values are the mean ± SEM of five independent experiments. ns: not significant; * *p* < 0.05.

Growing evidence has shown that melatonin plays a role in various pathological processes by binding to MT1 or MT2, two classical G-protein-coupled receptors [10]. Then, we applied luzindole, a melatonin receptor antagonist, to evaluate whether these receptors are involved in the anti-fibrotic effects of melatonin. We first performed qRT-PCR to detect the mRNA level of Col1α1 and Col3α1. As shown in Figure 2A,B, melatonin attenuated the TGF-β1-induced Col1α1 and Col3α1 formation in cultured lung fibroblasts along with TGF-β1 treatment, whereas this effect was alleviated by luzindole. Next, we examined which pathological process contributes to the anti-fibrotic action of melatonin. We found that melatonin mitigated TGF-β1-induced lung fibroblast migration (Figure 2C,D), which was abolished by luzindole. Intriguingly, melatonin alone did not exert an anti-fibrotic function compared to control. Moreover, we found that luzindole also significantly suppressed the anti-fibrotic function of melatonin, which was shown by the protein expression levels of collagen 1, Fn1, and α-SMA (Figure 2E,F).

**Figure 3:**
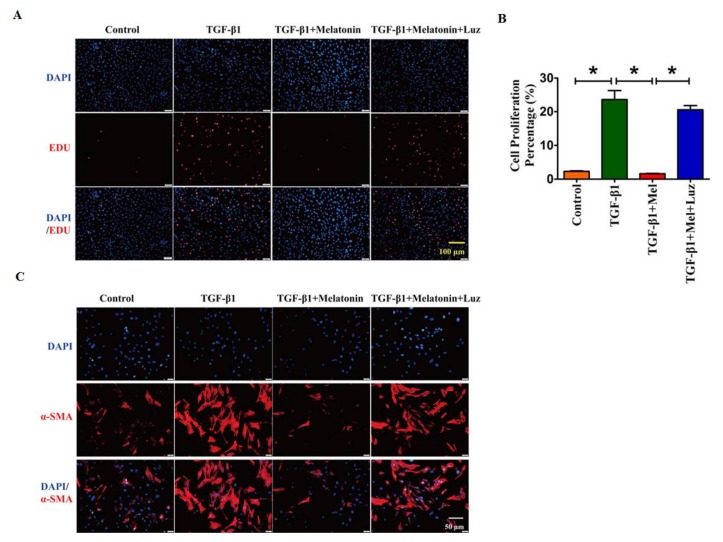
Luzindole suppresses the effect of melatonin in lung fibrogenesis along with the change of cell proliferation and myofibroblasts activation. (**A**) The analysis of EDU staining demonstrated the cell proliferation ability of lung fibroblasts with different treatments; (**B**) The statistical diagram of the EDU assay; (**C**) Immunostaining of α-SMA in lung fibroblasts demonstrated the inhibitory effect of luzindole. Values are the mean ± SEM of five independent experiments. * *p* < 0.05.

Additionally, we also discovered that melatonin suppressed the ability of lung fibroblasts proliferation driven by TGF-β1 (Figure 3A,B). Moreover, immunofluorescent staining of α-SMA indicated that melatonin attenuated the TGF-β1-induced fibroblast-myofibroblast transition, which was almost blunted by luzindole (Figure 3C). Therefore, these results indicated that melatonin receptors play a pivotal role in the process of pulmonary fibrosis.

### 2.3. Hippo/YAP1 Pathway Contributes to the Inhibitory Function of Melatonin during Pulmonary Fibrosis

**Figure 4:**
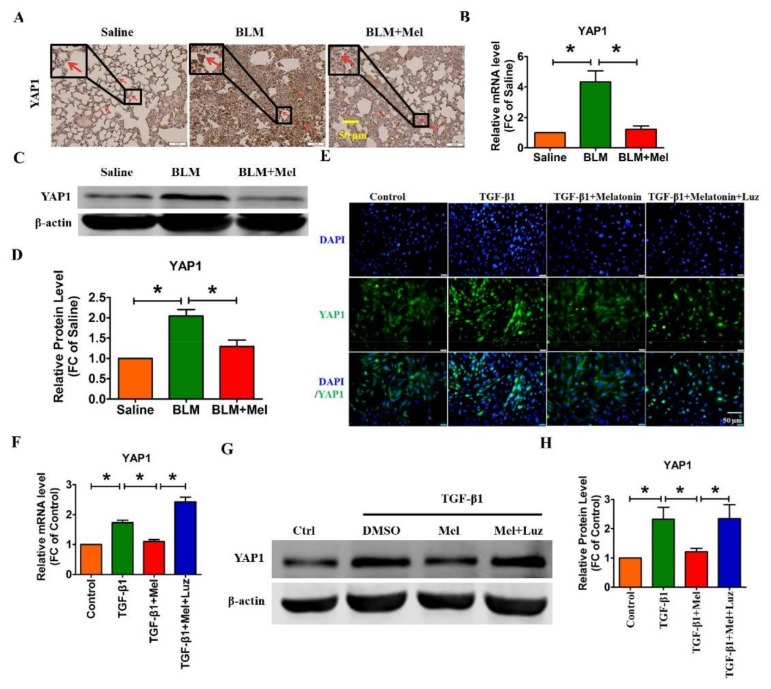
Dysfunction of YAP1 is involved in the anti-fibrotic effect of melatonin in BLM-treated mice. (**A**) The expression of YAP1 was down-regulated after treatment with melatonin, determined with immunohistochemical staining; red arrow indicated the expression of YAP1. (**B**) qRT-PCR analysis of the mRNA expression of YAP1 in BLM-mice. β-Actin mRNA acted as the internal control; *n* = 6 mice in each group. (**C**,**D**) Western blot analysis of YAP1 and the related statistical data are shown. (**E**) Immunofluorescence assay to determine the effect of melatonin and luzindole on the location of YAP1 in lung fibroblasts. qRT-PCR (**F**) and Western blot (**G**,**H**) assays were performed to evaluate the effect of melatonin and luzindole on the mRNA and protein level of YAP1. β-Actin served as the internal control. Values are the mean ± SEM of five independent experiments. * *p* < 0.05.

Accumulating evidence has shown that YAP1 participates in multiple physiological-fibrotic processes [20,21,22], and the activity of YAP1 is affected by numerous stimuli, such as GPCRs. Therefore, we assume that melatonin alleviates pulmonary fibrosis by inhibiting the functional role of YAP1 via binding to melatonin receptors [23]. First, we performed immunohistochemistry experiments to detect the differential expression of YAP1 in vivo and in vitro. As illustrated in Figure 4A, YAP1 was significantly up-regulated in BLM-induced lung fibrosis, which was abrogated by melatonin. Moreover, significant down-regulation of YAP1 mRNA and protein were observed in BLM-treated mice after treatment with melatonin (Figure 4B–D). Consistent with the in vitro results, the immunofluorescence assay revealed that melatonin eliminated the translocation of YAP1 from the cytoplasm into the cell nucleus induced by TGF-β1 in lung fibroblasts (Figure 4E). Meanwhile, the TGF-β1-induced up-regulation of YAP1 was remarkably mitigated by melatonin both at the mRNA and protein levels (Figure 4F–H). More importantly, the inhibitory effect of melatonin on YAP1 was abolished by luzindole (Figure 4E–H). These data demonstrate that melatonin attenuates lung fibrosis by inhibiting the expression and activation of YAP1 via binding to MT1/MT2.

### 2.4. Overexpression of YAP1 Abates the Anti-Fibrotic Effect of Melatonin in Lung Fibroblasts

**Figure 5:**
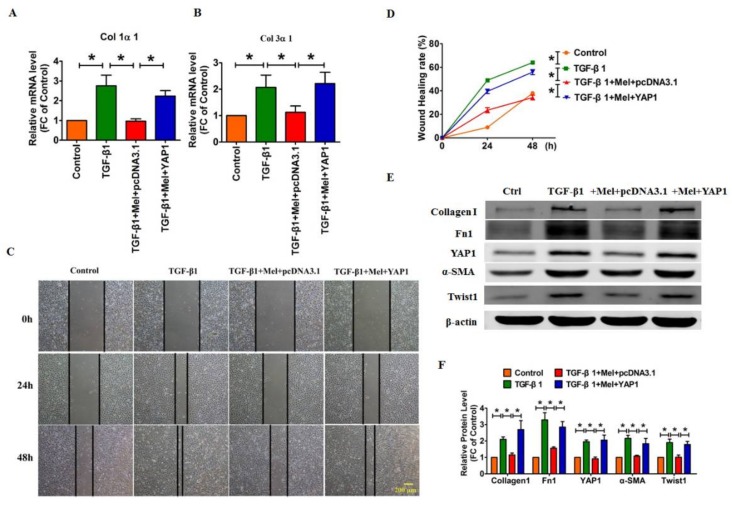
Forced expression of YAP1 abrogated the inhibitory effect of melatonin on production of collagen. (**A**,**B**) Real-time RT-PCR analysis showing that YAP1 overexpression blunted the inhibitory effects of melatonin on TGF-β1-induced up-regulation of Col 1α1 and Col 3α1 at the mRNA level. *n* = 6 cell batches. Representative photomicrographs showing that YAP1 restored the cell migration ability (**C**,**D**) compared with the TGF-β1+melatonin group. (**E**,**F**) Western blotting was used to determine the effect of YAP1 overexpression on the inhibitory effects of melatonin in TGF-β1-induced up-regulation of pro-fibrotic proteins, including collagen 1, Fn1, α-SMA and Twist1.Values are the mean ± SEM of five independent experiments. * *p* < 0.05.

Next, we performed a rescue experiment to investigate whether YAP1 mediated the anti-fibrotic effects of melatonin. As shown in Figure 5A,B, we found that overexpression of YAP1 mitigated the inhibitory effects of melatonin on Col1α1 and Col3α1 formation in lung fibroblasts. Moreover, melatonin abolished the TGF-β1-induced cell migration, whereas YAP1 had the opposite effect and blocked the anti-fibrotic effect of melatonin (Figure 5C,D). Meanwhile, Western blotting showed that melatonin inhibited the expression of several relevant fibrotic proteins, including Collagen1, Fn1, α-SMA and Twist1, whereas this effect was abolished when we reintroduced YAP1 in lung fibroblasts (Figure 5E,F).

**Figure 6:**
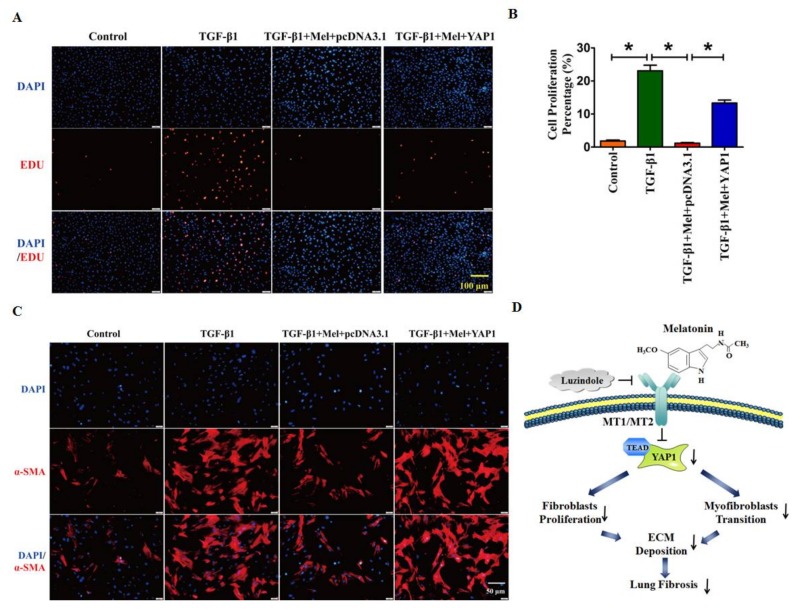
Overexpression of YAP1 reversed the suppression of cell proliferation and myofibroblasts activation. Microphotograph of EDU staining (**A**,**B**) and α-SMA positive staining (**C**) illustrated forced expression of YAP1 could rescue the anti-fibrotic effect of melatonin. (**D**) The schematic diagram of this study, melatonin alleviated lung fibrosis in vivo and in vitro via interacting with its receptor, and this anti-fibrotic effect was mediated by YAP1 inactivation. Values are the mean ± SEM of five independent experiments. * *p* < 0.05.

Moreover, the EdU staining assay and immunofluorescent staining revealed that melatonin abolished the TGF-β1-induced cell proliferation and myofibroblast activation, whereas these effects were effectively reversed with overexpression of YAP1(Figure 6A–C). These results suggested that melatonin alleviated TGF-β1-induced fibrogenesis by promoting YAP1 degradation, whereas exogenous YAP1 neutralized the impact of melatonin.

## 3. Discussion

In the present study, we characterized the inhibitory effect of melatonin in BLM-induced pulmonary fibrosis and clarified the potent molecular mechanisms to provide an ideal explanation of this effect. Moreover, melatonin alleviated lung fibrosis in vivo and in vitro by binding to its receptor, and this anti-fibrotic effect was mitigated by up-regulation and functional activation of YAP1 (Figure 6D). These findings suggest the therapeutic potential of melatonin for prevention and reversal of IPF.

Recently, emerging evidence has uncovered the significant role of melatonin in various pathophysiological processes [24]. However, the role and underlying mechanism of melatonin in lung fibrosis are not well understood [4], although some evidences have shown the effect of melatonin in lung fibrosis [25]. Arslan et al. found that melatonin inhibited BLM-induced lung fibrosis in rats by suppressing oxidative stress [26]. Consistent with this study, Yildirim et al. found that melatonin protected against lung fibrosis by suppressing catalase activity [13]. A recent study from Zhao et al. revealed that melatonin attenuates BLM-induced lung fibrosis by inhibiting epithelial-mesenchymal transition via inhibition of endoplasmic reticulum stress [27]. However, it is unclear whether melatonin exerts its anti-fibrotic role by regulating fibroblasts during lung fibrosis. As the most important effector cell, we found for the first time that melatonin inhibits the proliferation and migration of lung fibroblasts and mitigates fibroblast-myofibroblast transition during lung fibrosis. Meanwhile, melatonin has been shown to exert its biologic functions in a melatonin receptor-dependent or -independent manner [28,29]. In this study, we found that luzindole, an inhibitor of melatonin receptors, strikingly blocked the anti-fibrotic effect of melatonin, suggesting that melatonin alleviate pulmonary fibrosis via a melatonin receptor-dependent pathway. Interestingly, the melatonin receptor agonists Neu-P11 [30] and Tasimelteon [31] exerted a function similar to that of melatonin. Therefore, whether Neu-P11 or Tasimelteon can participate in the process of lung fibrosis and exhibit a more significant effect merits further investigation.

Recently, an increasing number of evidences have demonstrated that YAP1 contribute to the initiation and evolution of fibrotic diseases [32,33]. Whereas, subclinical function and mechanism of YAP1 in IPF have not been completely elucidated. Pan et al. discovered that Angiotensin II promote the collagen synthesis and cell proliferation in primary lung fibroblasts by increasing the YAP1 activity, ultimately leading to the progress of fibrosis [34]. Liu et al. demonstrated that YAP/TAZ mediate mechanosignaling-induced fibroblast activation and lung fibrosis [35]. Notably, in our previous studies, we illustrated that YAP1 contribute to the process of lung fibrosis. Overexpression of YAP1 enhanced the production of collagen and promoted the fibroblasts activation. In this study, we identified that forced expression of YAP1 abolished the inhibitory effect of melatonin in primary lung fibroblasts.

Recent studies have found that the Hippo pathway is regulated by G-protein-coupled receptor (GPCR) signaling [18]. Yu et al found that GPCRs regulate the activation of the Hippo pathway [36]. They proposed that G-coupled receptors stimulate Lats1/2 kinase and then increase YAP/TAZ phosphorylation. However, G12/13-, Gq/11-, and Gi/o-coupled receptors inhibited Lats1/2 kinase and promoted the dephosphorylation and nuclear localization of YAP1 [36]. Consistent with this study, they also confirmed that activation of mutated G_q_ and G_11_ promoted the occurrence and development of uveal melanoma by activating YAP [37]. In addition, multiple studies have reported that other GPCRs, such as angiotensin II type 1 receptor (AT1R) and G protein-coupled estrogen receptor (GPER), fulfill their function in a variety of physiological and pathological conditions by disrupting the Hippo pathway [38,39]. We supposed that melatonin inhibits lung fibrosis by regulating the activity and expression of the Hippo pathway through binding to melatonin receptors, which belong to the GPCR family. Interestingly, we found that melatonin inhibits the nuclear location and expression of YAP1, whereas this effect was nearly reversed by luzindole.

The present work revealed that melatonin alleviates lung fibrosis through YAP1 regulation. In a series of in vitro and in vivo experiments, we illuminated the critical role of YAP1 in the anti-fibrotic effect of melatonin. These findings indicate that administration of melatonin may be considered as a novel strategy for the treatment of lung fibrosis.

## 4. Materials and Methods

### 4.1. Experimental Pulmonary Fibrosis Model and Treatment

C57BL/6 mice (male; 6–8 weeks old) were obtained from Vital River Laboratory Animal Technology (Beijing, China). All animals were fed a chow diet and maintained in a 12-h light/12-h dark environment at 25 °C. In this work, the procedures for animal use were consistent with the regulations of the Ethics Committees of Harbin Medical University (No. 16520134, 1 March 2016) and conformed to the NRC Guide for the Care and Use of Laboratory Animals (2011, 8th ed.). To construct a pulmonary fibrosis model, bleomycin (BLM, Sigma-Aldrich Co., LLC, St. Louis, MO, USA) was injected intratracheally at a dose of 1.5 U/kg body weight.

Therefore, all the mice would be divided into three groups: Saline, BLM and BLM + Melatonin, there were six mice in each group. In BLM-induced pulmonary fibrosis animal model, melatonin (5mg/kg/d, Selleck, Shanghai, China) was intraperitoneally injected into mice for 21 days.

### 4.2. Isolation of Neonatal Mouse Lung Fibroblasts

Primary lung fibroblasts were isolated from the lungs of 1- to 3-day-old C57BL/6 mice. Neonatal mouse lung tissues were finely minced and placed together in 0.25% trypsin. After digestion for 90 min, the cell suspensions were centrifuged and resuspended in Dulbecco’s modified Eagle’s medium (DMEM, Gibco, NY, USA) supplemented with 10% fetal bovine serum (FBS, BI), 100 U/mL penicillin and 100 μg/mL streptomycin. The cell mixture was seeded into culture flasks and incubated for 6 to 8 h to allow for preferential attachment of fibroblasts. Non-adherent and weakly attached cells were removed, and anchorage-dependent cells were incubated at 37 °C with 5% CO_2_.

### 4.3. Procedures for Cell Transfection

For cell transfection, lung fibroblasts were incubated with serum-free medium for 6 h. Lung fibroblasts were transfected with YAP1 using Lipofectamine 2000 (Invitrogen, Carlsbad, CA, USA). YAP1, was separately mixed with Opti-MEM^®^ I Reduced Serum Medium (Gibco, NY, USA) for 5 min. Then, the two mixtures were combined and incubated at room temperature for 15 min. The combined mixture and Lipofectamine 2000 were added to the cell culture plate and incubated with cells at 37 °C with 5% CO_2_ for 36–48 h. In addition, in vitro experiments for lung fibroblasts transfection, Luzindole, a melatonin receptor inhibitor, was utilized at a dose of 100 nM/L.

### 4.4. Western Blot Analysis

Western blotting was performed as previously described [40]. Total protein samples from snap-frozen lung tissues and cultured lung fibroblasts were prepared and transferred to nitrocellulose membranes (Pall Life Science, Waltham, MA, USA). The following primary antibodies were used; anti-Collagen 1 (14695-1-AP, Proteintech, Rosemont, IL, USA), anti-Fn1 (15613-1-AP, Proteintech), anti-YAP1 (13584-1-AP, Proteintech), anti-α-SMA (Ab7817, Abcam, Cambridge, MA, USA), anti-Twist1 (WL0109, Wanleibio, Dalian, China) and anti-β-actin (60008-1-AP, Proteintech), which was used as an internal control. The immunoreactivity was detected using an Odyssey Infrared Imaging System. The intensity of each of the blot bands was measured with Odyssey 3.0 software (Gene Company Limited, Hongkong, China).

### 4.5. Masson’s Trichrome Staining

The lungs of mice were rapidly dissected, immersed in 4% paraformaldehyde for a week and stained with Masson’s trichrome according to the manufacturer’s instructions to assess the degree of fibrosis. The fibrotic areas of tissues were examined under high power and scored in a total of 10 random fields per specimen. Digitized images were analyzed with Image-Pro-Plus 6.0 software. (Media Cybernetics, Inc., Rockville, MD, USA)

### 4.6. Immunohistochemistry Staining

Immunohistochemistry (IHC) staining was carried out as previously described [41]. Mouse lung tissues were fixed with 4% paraformaldehyde for seven days, after being paraffin-embedded and sectioned. Primary antibodies against Fn1 and YAP1 were purchased from Proteintech (Rosemont, IL, USA). IHC was analyzed under a fluorescence microscope (DP80, Olympus, Tokyo, Japan).

### 4.7. SircolTM Soluble Collagen Assay

Collagen content assay was performed as previously described [19]. Total samples were extracted from lung tissues of mice treated with Saline, BLM or BLM + Mel. According to the manufacturers’ instruction of SircolTM Soluble Collagen Assay (Biocolor, Northern Ireland, UK), the detection of soluble collagen was followed. The results were analysed by GraphPad Prism 5.0. (GraphPad Software, Inc., La Jolla, CA, USA)

### 4.8. Immunofluorescence Staining

Lung fibroblasts were treated with TGF-β1 (10 ng/mL, PeproTech, Rocky Hill, NJ, USA) or melatonin (400 μmol/L) or transfected with YAP1/sh-YAP1 (2 μg) for 40 h. Then, the cells were fixed for 30 min in 4% paraformaldehyde at room temperature and permeabilized with 0.2% Triton X-100 in PBS. After treatment with blocking buffer (PBS containing 50% goat serum albumin), the cells were incubated overnight at 4 °C with anti-α-SMA (1:200; Abcam) and anti-Vimentin (1:500; Cell Signalling Technology, Beverly, MA, USA). One day later, the cells were washed three times and incubated for 2 h at room temperature in the dark with FITC-conjugated goat anti-mouse/rabbit antibody (1:500, Alexa Fluor 488; Life Technologies, Waltham, MA, USA). Nuclei were stained with DAPI (Roche Molecular Biochemicals, Inc., Pleasanton, CA, USA) for 6 min. Immunofluorescence was analyzed under a fluorescence microscope (Nikon 80i, Tokyo, Japan).

### 4.9. Scratch Wound-Healing Assay

Cells were seeded on 6-well plates and incubated overnight at 37 °C at 5% CO_2_. Artificial wounds were made using 10-μL pipette tips (0 h), to generate a gap in the confluent cell layer. Cells were washed with PBS and fed with either control (serum-free) medium or medium containing different treatment combinations (TGF-β1, melatonin and luzindole). Phase-contrast microscopy images were observed at different time points using a Nikon TS100 microscope (Tokyo, Japan).

### 4.10. EdU Fluorescence Staining

Fluorescence staining with 5-ethynyl-2′-deoxyuridine (EdU) was used to detect newly synthesized DNA in lung fibroblasts after the corresponding treatments. All steps of the manufacturer′s instructions for a Cell-Light EdU DNA cell proliferation kit (RiboBio, Guangzhou, China) were followed. Images were obtained with a fluorescence microscope (Nikon 80i, Tokyo, Japan).

### 4.11. Quantitative RT-PCR

Total RNA samples were extracted from lung tissues of mice or cultured lung fibroblasts using TRIzol (Invitrogen, Carlsbad, CA, USA). As demonstrated in our previous work [40], real-time RT-PCR was conducted on a 7500 Fast Real-Time PCR System (Applied Biosystems, Foster City, CA, USA) using Power SYBR^®^ Green Master Mix (Applied Biosystems). The comparative threshold cycle (*C*_t_) method was used to calculate the expression of fibrotic-related genes (Colα1, Colα3, et al.) β-Actin was used as the internal control.

### 4.12. Statistical Analysis

All data are presented as the mean ± SEM. One-way analysis of variance (ANOVA) followed by Dunnett’s test was used for multiple comparisons. A two-tailed value of *p* < 0.05 was considered to indicate a statistically significant difference. Statistical analyses were carried out using GraphPad Prism 5.0 and SPSS 14.0 software (Chicago, IL, USA).

## Abbreviations

IPF	Idiopathic pulmonary fibrosis
Mel	Melatonin
Luz	Luzindole
GPCR	G-protein-coupled receptor
BLM	Bleomycin
